# The role of perceived burdensomeness and thwarted belongingness in suicide disclosure likelihood: a social network perspective

**DOI:** 10.3389/fpsyt.2026.1877025

**Published:** 2026-08-11

**Authors:** Lauren E. McBride, Emma M. Parrish, Starla Thomas, Holly B. Shakya, Anthony Fulginiti, Michael L. Thomas, Eric Granholm, Charles Taylor, Colin Depp

**Affiliations:** 1 San Diego State University/University of California San Diego Joint Doctoral Program in Clinical Psychology, San Diego, CA, United States; 2 Department of Veterans Affairs Medical Center, San Diego, CA, United States; 3 Department of Psychiatry, University of California, San Diego, La Jolla, CA, United States; 4 Herbert Wertheim School of Public Health & Human Longevity Science, University of California, San Diego, La Jolla, CA, United States; 5 Graduate School of Social Work, University of Denver, Denver, CO, United States; 6 Department of Psychology, Colorado State University, Fort Collins, CO, United States

**Keywords:** affective disorders, perceived burdensomeness, social network, suicide disclosure, thwarted belongingness

## Abstract

**Background:**

Suicide prevention frequently hinges on disclosure of suicidal ideation (SI), yet half of suicide deaths are not preceded by disclosure. Interpersonal beliefs, including perceived burdensomeness and thwarted belongingness predict suicide risk, but little is known about how those beliefs influence SI disclosure likelihood. This study employed a social network approach to investigate the role of PB and TB in likelihood to disclose future SI.

**Methods:**

N = 111 adults (mean age=54.7 years, 78.2% women) with an affective or psychotic disorder completed a social network survey assessing network structure, quality, support, and likelihood to disclose future suicidal thoughts to each social network member. Participants were also assessed for perceived burdensomeness and thwarted belongingness, depressive symptoms, and current/lifetime SI. Correlations evaluated relationships among social network features, perceived burdensomeness and thwarted belongingness, and likelihood to disclose future suicidal thoughts. Multilevel mediation models evaluated whether perceived burdensomeness and thwarted belongingness explained the relationship between social network features and likelihood to disclose future suicidal thoughts, before and after adjusting for depression and current SI.

**Results:**

Social network structure, quality, and support were associated with perceived burdensomeness and thwarted belongingness. Perceived burdensomeness and thwarted belongingness were negatively correlated with likelihood to disclose future suicidal thoughts. Adjusting for depression and current SI, thwarted belongingness explained the relationship between closeness, satisfaction, and practical support received, and likelihood to disclose future suicidal thoughts (standardized indirect effects *β* ≈.19 −.45). Perceived burdensomeness did not account for the relationship between social network features and likelihood to disclose future suicidal thoughts after adjusting for depression and SI.

**Discussion:**

Thwarted belongingness, but not perceived burdensomeness, explained the relationship between social network features and likelihood to disclose future suicidal thoughts after adjusting for depression and SI. Our findings highlight the independent role of thwarted belongingness in anticipated suicide disclosure decisions and suggest that interventions that strengthen social networks could improve thwarted belongingness and suicide disclosure.

## Background

Suicide is a leading cause of death worldwide, with approximately 727,000 individuals dying by suicide in 2021 (1). Disclosure of suicidal ideation is a key initial step to initiating interventions, yet only 50-60% of individuals with suicidal ideation disclose it to others (2). Disclosure often occurs within trusted social relationships, and relationship factors such as closeness or expected emotional support can influence one’s decision to disclose (3–5). The Interpersonal Theory of Suicide posits that perceived burdensomeness (PB), or the feeling of being a burden on others, and thwarted belongingness (TB), or feeling that one does not belong with others, are strong predictors of suicidal ideation and behavior (6). Due to the link between these constructs, suicidal ideation, and perceptions about social relationships, PB and TB may also impact an individual’s decision to disclose suicidal ideation to others. A better understanding of how PB and TB influence suicide disclosure could inform whether interventions targeting these constructs might also be beneficial for increasing suicide disclosure likelihood.

Extant literature on the relationship of PB and TB to suicide disclosure is limited. Most prior work has evaluated if past disclosure experiences, whether positive or negative, influence current feelings of PB or TB (7, 8). There is evidence that PB and TB are negatively correlated with suicide-related disclosure (7). The relationship between past disclosure and PB and TB has been found to be mediated by perceived social support (8) and whether family members reacted positively following disclosure of suicidal ideation (7). To our knowledge, no prior study has examined whether PB and TB influence future likelihood to disclose suicidal ideation.

Given the inherent interpersonal nature of PB and TB, these constructs are deeply tied to social connection. Social connection is a complex and multidimensional construct, often described by structural (e.g., network size, contact frequency), quality (e.g., relationship closeness, satisfaction), and functional aspects (e.g., perceived emotional and practical support) (9). Social network measures, in which individuals nominate close contacts and answer specific questions about each person, are useful to capture the overall social network composition and the multidimensional aspects of each relationship within an individual’s network (10). Social network measures have been previously utilized in suicide disclosure research (3, 4, 11), and prior literature suggests that relationship quality (closeness) and function (perceived emotional and practical support) are associated with suicide disclosure likelihood (3–5). Moreover, multiple studies have found that higher perceived social support was associated with lower PB and/or TB and that greater contact frequency is linked to lower TB (6, 12). However, no studies have evaluated whether PB or TB serve as mechanisms that link structure, quality, and function of social networks to disclosure.

To address these gaps, the present study employed an adapted social network measure to investigate relationships between PB and TB; structural, functional, and qualitative relationship features and the likelihood of disclosing future suicidal thoughts in a transdiagnostic sample of N = 111 adults. We assessed whether PB and TB are associated with social network features and likelihood to disclose suicidal thoughts. Based on pre-existing literature (3–5), we hypothesized that closeness, satisfaction, perceived emotional support, and perceived practical support would predict greater likelihood of disclosing future suicidal thoughts, while structural factors (network size, contact frequency, and network density) would not be associated with the likelihood to disclose future suicidal thoughts. We also hypothesized that PB and TB would be negatively correlated with likelihood to disclose future suicidal thoughts (7).

We conducted two additional sets of exploratory analyses, for which no *a priori* hypotheses were specified. First, because depression, PB, and TB can all reduce the likelihood to disclose future suicidal thoughts (13–17), we evaluated which associations were significant after adjusting for depression. Second, we assessed whether PB and TB account for associations between social network features and future suicidal thought disclosure likelihood.

## Materials and methods

### Participants

The present manuscript utilizes datasets from two ongoing studies researching transdiagnostic suicide help-seeking processes, one focused on older adults and the other recruiting participants across the lifespan. Because the studies assessed similar research questions and had comparable methods and measures, the datasets were combined for analyses. Data from these samples were previously reported (5) but did not include PB and TB or mediation analyses. The studies recruited participants in San Diego through referrals from University of California, San Diego clinics, social media, and community flyers. The inclusion age range was 18–65 years for the first study (Study 1; NIMH R01MH130396) and 60 years or older for the second study (Study 2; NIMH R01MH132112). For studies 1 and 2, individuals were required to have a diagnosis of a depressive disorder, an anxiety disorder, or post-traumatic stress disorder, which was confirmed by the Structured Clinical Interview for the DSM-5 (SCID-5, Research version) (18). Study 1 also included individuals with a psychotic disorder, confirmed by Modules B & C of the SCID-5. The SCID-5 was administered to all participants by trained research staff to assess study eligibility. The exclusion criteria for both studies were as follows: substance use disorder within the past 3 months, besides cannabis or tobacco; history of head trauma with a loss of consciousness>15 minutes; a neurodegenerative disorder (e.g., dementia); an intellectual disability; sensory impairments precluding assessments (e.g., visual limitations); or if they could not pass an informed consent capacity assessment (19). A total of 188 individuals participated across both studies (Study 1 n=72, Study 2 n=116). This analysis included participants who reported that they could hypothetically anticipate having future suicidal thoughts (Study 1 n=47, Study 2 n=64, total n=111). Both studies were reviewed and approved by the University of California, San Diego Institutional Review Board, and formal ethical approval was granted for each study prior to data collection. All participants provided written informed consent.

### Measures

Data presented here were collected at baseline across both studies and are cross-sectional. All measures were administered in English. Demographic characteristics (age, gender, race, ethnicity) were collected via self-report. Across analyses, nonbinary and female participants were combined due to the small number of nonbinary participants (n=1), and race was dichotomized into White and non-White due to small cell sizes. Ethnicity was captured dichotomously (Hispanic or Latino/a/x/e/or not Hispanic or Latino/a/x/e). Current and lifetime suicidal ideation were captured via the Columbia Suicide Severity Rating Scale, which was administered by trained research staff (C-SSRS; Baseline/Screening version) (20). Participants also reported whether they had a suicide attempt in their lifetime within the C-SSRS. Current suicidal ideation was defined by the presence of passive (wish to go to sleep and not wake up) or more severe thoughts of suicide in the last month (a score of 1 or greater), while lifetime suicidal ideation was defined by the presence of passive or more severe suicidal thoughts at any point in the participant’s lifetime (a score of 1 or greater). For analyses, current and lifetime suicidal ideation were dichotomized (1= suicidal ideation present, 0=no suicidal ideation). Depression symptoms within the past two weeks were captured using the Patient Health Questionnaire-9 (PHQ-9) (21), which is a widely utilized self-report measure. The PHQ-9 demonstrated excellent internal consistency within this sample (*α* = 0.91).

#### Social network survey

We adapted a self-report social network survey from the Gallup longitudinal, egocentric social network survey (22). Questions querying likelihood to disclose future suicidal thoughts were adapted from social network interviews previously utilized by Fulginiti et al. (4, 11, 23). Item-level responses from a pilot sample were previously reported (5). Participants used three prompts to nominate up to 12 people: individuals they spend their free time with, individuals they discuss personal matters with, and individuals they considered a friend in the last three months. They could nominate a maximum of four individuals per prompt category (for a total of 12 possible independent people), and the same individuals could be named across multiple categories. Analyses are based on participants’ responses to six Likert-type questions that they answered about each network member.

#### Structural network features

We derived three structural network variables: network size, network density, and contact frequency. Network size is the number of unique individuals each participant nominated. Network density is the proportion of connections within a network out of all possible connections (range=0-1). For contact frequency, participants reported how frequently they interact with each network member via phone, text, email, and in person on a scale from 0 (never) to 4 (every day or nearly every day). We generated a sum score across modalities for each network member within each participant to calculate contact frequency (range=0-16).

#### Quality network features

We derived two quality network variables: relationship closeness (“How close do you feel to [network member]?”) and relationship satisfaction (“How satisfied are you with your relationship with [network member]?”. Answer choices were on a 7-point scale (1=not close/satisfied at all, 7=extremely close/satisfied).

#### Functional network features

We derived two functional network variables: emotional support (“To what extent can you count on [network member] when you need emotional help or support?”) and practical support (“To what extent can you count on [network member] when you need practical help in everyday life?”). Answer choices were on a 7-point scale (1=not at all, 7 =very much).

#### Likelihood to disclose future suicidal thoughts

The outcome variable in all models was likelihood to disclose future suicidal thoughts (“If you were to ever think about killing yourself in the future, how likely is it that you would talk to [network member] about these thoughts?”). Answer choices were on a 7-point scale (1=extremely unlikely, 7=extremely likely).

#### Perceived burdensomeness and thwarted belongingness

PB and TB were measured using the Interpersonal Needs Questionnaire (INQ), which is a 15-item self-report measure that is widely used to assess interpersonal constructs related to the Interpersonal Theory of Suicide (6). The measure includes a series of statements about participants’ thoughts about themselves and others, and participants are prompted to respond with how much they agree with each statement on a 7-point scale (1=not at all true for me, 7=very true for me). The measure has two subscales: PB, which includes 6 questions, and TB, which includes 9 questions. PB subscale scores range from 6 to 42, and TB subscale scores range from 9 to 63. Higher scores indicate greater PB and TB. INQ subscales demonstrated excellent internal consistency within this sample (PB scale *α* = 0.91; TB scale *α* = 0.91).

### Analysis

Analyses were conducted using *R* version 4.4.2 (24). Descriptive statistics were computed for demographic and social network variables, as well as for PB and TB scores. Data were inspected for non-normality visually using histograms. Pearson’s correlations were calculated between social network features, PB and TB, and likelihood to disclose future suicidal thoughts. For any significant bivariate correlations, partial correlations were computed to assess whether the relationship was still significant after adjusting for depression. Correlation magnitudes were interpreted using the guidelines by Akoglu (25). A Benjamini-Hochberg false discovery rate correction (FDR) was applied to adjust for multiple comparisons for correlations (26). Families of tests were defined by social network feature domain (structure, quality, or function) and correlate (PB, TB, or likelihood to disclose future suicidal thoughts).

**Figure 1:**
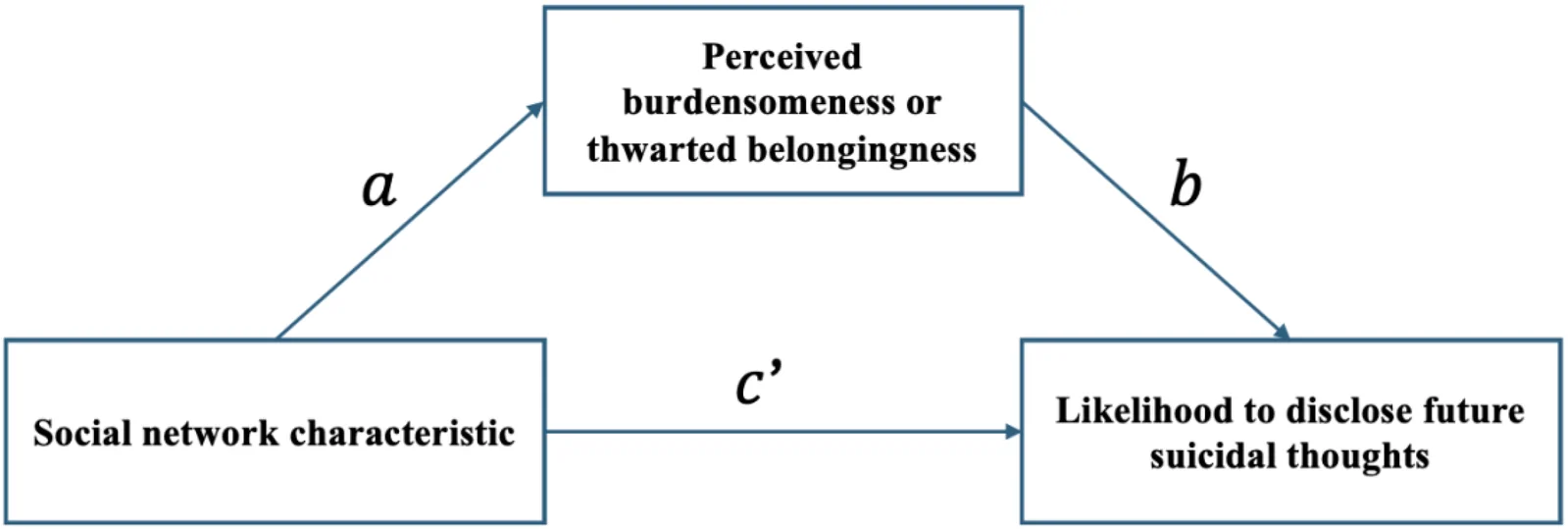
Path model diagram. **(a)** effect of social network characteristic on perceived burdensomeness or thwarted belongingness; **(b)** effect of social network characteristic on likelihood to disclose future suicidal thoughts; **(c')** direct effect of social network characteristic on likelihood to disclose future suicidal thoughts after accounting for mediator.

Exploratory mediation analyses were then conducted using a mixed-effects approach. Social network surveys produce a one-to-many data structure, with multiple participant-network member pairs nested within participants. To account for data structure, multilevel structural equation modeling was utilized to examine both within-person and between-person associations (27). At the within-person level (level 1), the models tested whether social network features predicted the likelihood to disclose future suicidal thoughts to a given network member (530 total network members nominated). At the between-person level (level 2), the models evaluated whether PB and TB mediated the association between average social network features and the average likelihood to disclose future suicidal thoughts (111 total participants at this level). Models were estimated using the lavaan *R* package (28). Due to the non-normality of the data, all models were estimated using maximum likelihood with robust standard errors (MLR). To handle missing data, full information maximum likelihood (FIML) was utilized, which leverages all available information to produce unbiased parameter estimates (29). Standard errors were estimated using cluster-robust sandwich estimators under MLR to account for within-person clustering. Each model included one social network feature as a predictor, PB or TB as a mediator, and likelihood to disclose future suicidal thoughts as the outcome variable (see **Figure 1**). Confidence intervals for indirect effects were calculated using the delta method, which was implemented using the lavaan *R* package. To reduce the total number of analyses, this set of analyses only included social network features that were significantly correlated with the likelihood to disclose future suicidal thoughts.

Prior to generating multi-level path models, linear regression models were estimated to determine which potential covariates (age, race, ethnicity, gender, Study 1 versus Study 2) significantly predicted the endogenous variables. Covariates significant at the *p<*.05 level were included in all multi-level path models.

For path models that evidenced a significant indirect effect, depression was added as a covariate in a second iteration of the model to evaluate whether PB and TB explained the relationship between social network features and likelihood to disclose future suicidal thoughts over and above depression. Separate models were also generated with presence of current suicidal ideation (yes or no) as a covariate.

All multi-level path models were just identified (*df* = 0), so global fit indices (e.g., X^2^, RMSEA, SRMR, CFI) were not interpretable. Model interpretation instead focused on statistical and practical significance of indirect and direct effects. All coefficients reported are standardized. Statistical tests were two-tailed with *p<*.05.

## Results

### Sample characteristics

The sample included 111 participants ages 18-82, and most participants identified as white and female. Participants contributed a total of 530 social network connections, and the average network size was 4.8 people (SD = 1.9, range=1-10). The most common psychiatric diagnosis was major depressive disorder, and 37.8% of the sample (n=42) endorsed having suicidal ideation in the past month. Most participants (83.8% of the sample, n=93) reported past SI and 30.5% (N = 32) reported a past suicide attempt. Demographic data and descriptive statistics for social network features are shown in **Table 1**. On the INQ, the average PB score was 12.5 (SD = 7.7, Range=6-37), and the average TB score was 31.1 (SD = 13.0, Range=9-58). The average PHQ-9 score was 10.7 (SD = 6.3, Range=0-27), which is consistent with moderate symptoms of depression (21).

**Table 1:** Demographic characteristics and social network features.

Characteristic	Participants (n=111)
Age, years, M(SD), Range	54.7 (19.1), 18 – 82
Gender, n (%)	
Male	23 (20.9%)
Female	86 (78.2%)
Non-binary	1 (0.9%)
Race, n (%)	
White	75 (68.2%)
Black or African American	9 (8.2%)
Asian	13 (11.8%)
More than one race or other	9 (8.2%)
Unknown	4 (3.6%)
Ethnicity, n (%)	
Hispanic or Latino/a/x/e	19 (17.3%)
Not Hispanic or Latino/a/x/e	91 (82.7%)
Current and past suicide risk	
Current suicidal ideation, n (%)	42 (37.8%)
Past suicidal ideation, n (%)	93 (83.8%)
Past suicide attempt, n (%)	32 (30.5%)
Primary psychiatric diagnosis, n (%)	
Major depressive disorder without psychosis	63 (63.0%)
Anxiety disorder	23 (23.0%)
Post-traumatic stress disorder	2 (2.0%)
Schizophrenia spectrum disorder	11 (11.0%)
Major depressive disorder with psychosis	1 (1.0%)
*Social network features*	
Structural social network features, M(SD), Range	
Network size	4.8 (1.9), 1 – 10
Contact frequency	8.2 (3.3), 1 – 16
Network density	0.68 (0.29), 0 – 1
Quality social network features, M(SD), Range	
Relationship closeness	5.4 (1.3), 1 – 7
Relationship satisfaction	5.4 (1.5), 1 – 7
Functional social network features, M(SD), Range	
Perceived practical support	5.1 (1.8), 1 – 7
Perceived emotional support	4.9 (1.9), 1 – 7
Likelihood to disclose suicidal thoughts, M(SD), Range	3.7 (2.2), 1 – 7

### Bivariate correlations: social network features, PB and TB, and likelihood to disclose future suicidal thoughts

PB and TB were significantly negatively correlated with likelihood to disclose future suicidal thoughts (PB *r* = −.36, *p* <.001; TB *r* = −.50, *p* <.001). Partial correlations that adjusted for depression remained significant (PB *r* = −.25, *p* = .010; TB *r* = −.43, *p* <.001). PB and TB were also significantly correlated with each other (*r* = .50, *p* <.001).

Correlation coefficients between social network variables and PB and TB are shown in **Table 2**. Among structural social network variables, greater network size was weakly correlated with lower TB before and after adjusting for depression, and greater contact frequency was weakly correlated with both lower PB and lower TB before and after adjusting for depression. Network density was not correlated with PB or TB. Among quality variables, closeness and satisfaction were weakly negatively correlated with PB and moderately negatively correlated with TB. After adjusting for depression, satisfaction was no longer correlated with PB but remained significantly correlated with TB. Among functional social network variables, emotional and practical support received were weakly negatively correlated with PB and moderately negatively correlated with TB before and after adjusting for depression.

**Table 2:** Correlations between social network variables and perceived burdensomeness and thwarted belongingness, adjusted for depressive symptoms.

*Domain*	*Variable*	Perceived burdensomeness	Perceived burdensomeness adjusting for depression	Thwarted belongingness	Thwarted belongingness adjusting for depression
Structural	Network size	r=-.05, *p=*.621	–	r=-.36, *p<*.001**	r=-.32, *p=*.001*
	Network density	r=-.14, *p=*.157	–	r=-.10, *p=*.297	–
	Contact frequency	r=-.20, *p=*.035*^†^	r=-.25, *p=*.009*	r=-.18, *p=*.061	–
Quality	Closeness	r=-.25, *p=*.008*	r=-.26, *p=*.007*	r=-.32, *p=*.001*	r=-.33, *p<*.001**
	Satisfaction	r=-.26, *p=*.006*	r=-.12, *p=*.212	r=-.54, *p<*.001**	r=-.47, *p<*.001**
Functional	Perceived emotional support	r=-.28, *p=*.003*	r=-.25, *p=*.009*	r=-.48, *p<.*001**	r=-.48, *p<*.001**
	Practical support received	r=-.29, *p=*.003*	r=-.26, *p=*.006*	r=-.36, *p<.*001**	r=-.34, *p<.*001**

*Significant at p<.05; **significant at p<.001; ^†^non-significant after FDR correction. Correlations adjusting for depression were not generated for variables that were not significantly correlated with perceived burdensomeness or thwarted belongingness.

Correlation coefficients between social network variables and likelihood to disclose future suicidal thoughts are shown in **Table 3**. All quality (closeness, satisfaction) and functional (perceived practical support, perceived emotional support) features significantly predicted greater likelihood of disclosing future suicidal thoughts before and after adjusting for depression. Among structural features, contact frequency, but not network size or network density, predicted greater likelihood of disclosing future suicidal thoughts before and after adjusting for depression. Correlation coefficients ranged in magnitude from weak to moderate.

**Table 3:** Correlations between social network variables and likelihood to disclose suicidal thoughts, adjusted for depressive symptoms.

*Domain*	*Variable*	Likelihood to disclose suicidal thoughts	Likelihood to disclose suicidal thoughts, adjusting for depression severity
Structural	Network size	r=.06, *p=*.507	–
	Network density	r=.18, *p=*.063	–
	Contact frequency	r=.33, *p<*.001**	r=.35, *p<*.001**
Quality	Closeness	r=.42, *p<*.001**	r=.42, *p<*.001**
	Satisfaction	r=.28, *p=*.003*	r=.21, *p=*.032*
Functional	Perceived emotional support	r=.54, *p<*.001**	r=.53, *p<*.001**
	Perceived practical support	r=.29, *p=*.002*	r=.26, *p=*.006*

*Significant at p<.05; **significant at p<.001; correlations adjusting for depression were not generated for variables that were not significantly correlated with likelihood to disclose suicidal thoughts.

### Multi-level path models: social network features, PB and TB, and likelihood to disclose future suicidal thoughts

Of potential covariates (race, gender, ethnicity, age, Study 1 versus Study 2), only ethnicity was significantly related to likelihood to disclose future suicidal thoughts (*b* = 1.46, *p* = .0001), with individuals who identified as Hispanic or Latino/a/x reporting greater likelihoods to disclose future suicidal thoughts. As such, ethnicity was included as a covariate in all multi-level path models. No covariates significantly predicted PB or TB. Direct within-person effects between social network features and likelihood to disclose future suicidal thoughts are shown below. These are followed by between-person effects grouped by mediator (PB or TB).

### Within-person effects of social network features on likelihood to disclose future suicidal thoughts

At the within-person level, participants endorsed higher likelihood of disclosing future suicidal thoughts to network members with whom they had more frequent contact (*β* = 0.15, *p* = .004), closer network members (*β* = 0.43, *p* <.001), network members who provided them with more emotional support (*β* = 0.48, *p* <.001) and practical support (*β* = 0.23, *p* <.001), and to network members with whom they reported greater relationship satisfaction (*β* = 0.31, *p* <.001). These findings were consistent with between-person associations tested in correlation analyses.

### Between-person indirect effects of perceived burdensomeness and thwarted belongingness

#### Perceived burdensomeness

There was a significant indirect effect of satisfaction across relationships on likelihood to disclose future suicidal thoughts through PB (*β* = .14, 95% *CI* [0.02, 0.25], *SE* = 0.11, *p* = .029). After accounting for PB, the relationship between satisfaction and likelihood to disclose future suicidal thoughts was non-significant, consistent with full mediation. There was also a significant indirect effect of perceived practical support on likelihood to disclose future suicidal thoughts through PB (*β* = .15, 95% *CI* [0.03, 0.28], *SE* = 0.10, *p* = .026). The relationship between perceived practical support and likelihood to disclose future suicidal thoughts was non-significant after adjusting for PB, which was consistent with full mediation. There were no significant indirect effects of contact frequency, closeness, or perceived emotional support on likelihood to disclose future suicidal thoughts through PB. Path coefficients for all models are shown in **Table 4**.

**Table 4:** Path coefficients for mediation analyses.

			Perceived burdensomeness				Thwarted belongingness			
Domain	Predictor	Path	Standardized estimate	SE	p	Mediation description	Standardized estimate	SE	p	Mediation description
Structure	Contact frequency	a: contact frequency -> PB or TB	-0.39	0.93	.026	None	-0.28	1.41	.079	None
		b: PB or TB -> disclosure likelihood	-0.31	0.02	.006		-0.49	0.01	<.001	
		c’: contact frequency -> disclosure likelihood (adjusting for PB or TB)	0.27	0.17	.123		0.25	0.14	.087	
Quality	Closeness	a: closeness -> PB or TB	-0.39	1.90	.023	None	-0.51	2.37	<.001	Full
		b: PB or TB -> disclosure likelihood	-0.30	0.02	.001		-0.47	0.01	<.001	
		c’: closeness -> disclosure likelihood (adjusting for PB or TB)	0.33	0.27	.010		0.20	0.31	.169	
	Satisfaction	a: satisfaction -> PB or TB	-0.36	1.36	.016	Full*^†^	-0.72	1.83	<.001	Full
		b: PB or TB -> disclosure likelihood	-0.37	0.02	<.001		-0.73	0.02	<.001	
		c’: satisfaction -> disclosure likelihood (adjusting for PB or TB)	0.12	0.22	.361		-0.27	0.30	.126	
Function	Emotional support	a: emotional support -> PB or TB	-0.43	1.25	.005	None	-0.67	1.71	<.001	Full*
		b: PB or TB -> disclosure likelihood	-0.23	0.02	.022		-0.36	0.02	.019	
		c’: emotional support -> disclosure likelihood (adjusting for PB or TB)	0.46	0.21	.001		0.33	0.27	.056	
	Practical support	a: practical support -> PB or TB	-0.45	1.07	.001	Full*^†^	-0.51	1.60	<.001	Full
		b: PB or TB -> disclosure likelihood	-0.34	0.02	.002		-0.53	0.01	<.001	
		c’: practical support -> disclosure likelihood (adjusting for PB or TB)	0.17	0.24	.302		0.05	0.22	.725	

*Mediation no longer present after adjusting for depression (path coefficients can be found in **Supplementary 1**, **3**).

† Mediation no longer present after adjusting for current suicidal ideation (path coefficients can be found in **Supplementary 2**, **4**).

For models with significant indirect effects, two additional models were estimated: one with depression symptom severity as a covariate and the other with presence of current suicidal ideation as a covariate. After adjusting for depression, the indirect effects of both satisfaction and perceived practical support on likelihood to disclose future suicidal thoughts through PB were no longer significant (see **Supplementary 1**). The models were also no longer significant after adjusting for current suicidal ideation (see **Supplementary 2**).

#### Thwarted belongingness

There was evidence of an indirect relationship between four social network features (satisfaction, closeness, perceived practical support, perceived emotional support) and the likelihood to disclose future suicidal thoughts through TB. Specifically, there were significant indirect effects of satisfaction, (*β* = 0.53, 95% *CI* [0.24, 0.81], *SE* = 0.27, *p* = .001), closeness (*β* = 0.24, 95% *CI* [0.07, 0.41], *SE* = 0.18, *p* = .006), perceived practical support (*β* = 0.27, 95% *CI* [0.10, 0.44], *SE* = 0.13, *p* = .003), and perceived emotional support (*β* = 0.24, 95% *CI* [0.27, 0.45], *SE* = 0.16, *p* = .023) on likelihood to disclose future suicidal thoughts through TB. Direct effects of satisfaction, closeness, perceived practical support, and perceived emotional support on likelihood to disclose future suicidal thoughts were non-significant after adjusting for TB (see **Table 4**), which is consistent with full mediation. There was not a significant indirect effect of contact frequency on likelihood to disclose future suicidal thoughts through TB.

After adjusting for depression, the indirect effects of closeness, perceived practical support, and satisfaction on likelihood to disclose future suicidal thoughts through TB remained significant. The indirect effect of perceived emotional support was no longer significant (see **Supplementary 3**). After adjusting for current suicidal ideation, indirect effects through TB remained significant in all models (see **Supplementary 4**).

## Discussion

This study sought to investigate the role of the Interpersonal Theory of Suicide constructs perceived burdensomeness and thwarted belongingness in predicting likelihood to disclose future suicidal thoughts to social network members. Perceived burdensomeness and thwarted belongingness were negatively related to likelihood of disclosing future suicidal thoughts. Notably, these associations were significant after adjusting for depression symptom severity. In path models, thwarted belongingness fully explained associations between closeness, satisfaction, and perceived practical support with likelihood to disclose future suicidal thoughts, even when adjusting for depression and current suicidal ideation. Perceived burdensomeness fully explained the relationship between perceived practical support and satisfaction with likelihood to disclose future suicidal thoughts, but these effects were not evident after adjusting for depression and current ideation. Taken together, these findings indicate that thwarted belongingness in particular may be an important pathway linking social connectedness with the likelihood to disclose future thoughts of suicide.

Prior research has evaluated the link between functional dimensions of social connection (i.e., perceived social support) and perceived burdensomeness and thwarted belongingness. Namely, Van Orden et al. (30) established that thwarted belongingness, but not perceived burdensomeness, relates to perceived social support, as well as related constructs, such as loneliness (30). Relatedly, more recent literature has found that both perceived burdensomeness and thwarted belongingness predicted lower perceived social support in a sample of individuals with early psychosis (31). Our study expands past literature by assessing how perceived burdensomeness and thwarted belongingness relate to social connectedness more broadly, which encompasses the related but distinct structural (objective number of social contacts or frequency of contact), quality (relationship closeness, satisfaction), and functional (perceived emotional and practical support) dimensions (9). Consistent with prior research, thwarted belongingness was negatively associated with structural, quality, and functional dimensions of social connectedness. Perceived burdensomeness was negatively associated with quality and functional dimensions, which aligns with work conducted by Moe et al. (31) but diverges from the findings of Van Orden et al. (30). In terms of structural features, thwarted belongingness was moderately negatively associated with network size, which aligns with prior research linking being around others and lower thwarted belongingness (12). After an FDR correction, no structural relationship features predicted perceived burdensomeness. While quality and functional dimensions of social connection appear important to both thwarted belongingness and perceived burdensomeness, structural indicators of social connectedness are uniquely related to thwarted belongingness, even when accounting for the effects of depression. The importance of subjective aspects of social connection (quality and functional dimensions) relative to objective social connectedness (structural dimensions) in predicting perceived burdensomeness and thwarted belongingness mirrors a well-established distinction in the broader social connection literature, which finds that subjective states such as loneliness are more predictive of a wide range of psychological outcomes than objective measures, such as social contact frequency (32, 33).

Past literature assessing the link between suicidal thought disclosure and perceived burdensomeness and thwarted belongingness is limited, though existing research has found that disclosure of past suicidal ideation predicts lower levels of both interpersonal beliefs (7). Our findings expand this literature by evaluating how perceived burdensomeness and thwarted belongingness relate to the likelihood of disclosing future suicidal thoughts. Consistent with past research, we found that both interpersonal beliefs predicted lower likelihood of disclosing future suicidal thoughts, even when adjusting for depression symptoms. While perceived burdensomeness and thwarted belongingness are closely linked with more severe psychological distress (34–36), this finding indicates that they may be unique and potentially important constructs implicated in not only the development of suicidal desire, but also decisions to disclose future suicidal thoughts.

These findings offer new insights into how social network features may relate to the likelihood of disclosing suicidal thoughts, extending prior work linking these constructs (3, 4). Within the Interpersonal Theory of Suicide (6), thwarted belongingness was originally conceptualized as a critical factor in the development of suicidal desire. Our results apply this conceptualization to disclosure of suicidal thoughts, such that perceptions about the availability and quality of relationships shape an individual’s perceptions of whether they socially belong, which in turn relate to disclosure of suicidal thoughts with others. Depressive symptoms are associated with a lower likelihood of suicide disclosure (17, 37), a phenomenon that has been labelled the “help negation effect” (38). Our findings extend the help negation effect literature and suggest that beyond depression, thwarted belongingness may play a unique and important role in how the positive influence of social connectedness on likelihood to disclose future suicidal thoughts may be attenuated at lower levels of belonginess. Perceived burdensomeness, on the other hand, did not explain the link between social network features and likelihood to disclose future suicidal thoughts after adjusting for depression or current suicidal ideation. This finding aligns with the theoretical conceptualization of perceived burdensomeness as encompassing generalized negative perceptions of the self, which may be difficult to disentangle from depression (6).

These findings have potential implications for suicide prevention and interventions targeting increased suicide-related disclosure. There are several existing psychosocial interventions targeting thwarted belongingness (e.g. (39–42),), which may be useful in increasing the likelihood of suicidal thought disclosure. Relationship-targeted interventions that influence quality and functional aspects of social connections (see (43) for a review), including those tailored for suicide prevention (44), may improve thwarted belongingness and perceived burdensomeness, and potentially increase subsequent disclosure. Finally, assessment of an individual’s likelihood to disclose future suicidal thoughts to specific network members could be added to existing suicide prevention interventions that rely upon suicide disclosure, such as safety planning where individuals list contacts from their social networks for distraction or support during crisis (45).

There are several limitations. Data were cross-sectional, and directional influences among constructs would require a longitudinal study design. For example, it may be that social network features explain the link between perceived burdensomeness and thwarted belongingness and likelihood to disclose future suicidal thoughts or that disclosure influences perceptions of burdensomeness and belongingness. Our study focused on likelihood to disclose future suicidal thoughts rather than actual disclosure, and not all participants had current thoughts of suicide. However, prior research has found that help-seeking intentions for suicidal thoughts predict future help-seeking behavior (46), and a recent scoping review found that numerous studies have linked mental health help-seeking intentions to future behavior (47), suggesting that the intention to disclose suicidal thoughts is predictive of future disclosure behaviors. The present study focused specifically on the role of social connectedness. However, prior literature has found that other types of connectedness, including religion, the broader community, or the natural environment, may be protective against suicide risk (48, 49). Future research should assess whether these other forms of connectedness relate to suicide disclosure processes. Another limitation is that the sample was predominantly white and female, limiting the generalizability to more racially diverse populations and males; moreover, the sample size did not allow for evaluating moderated mediation such as across demographic strata. Prior literature has found demographic differences in perceived burdensomeness and thwarted belongingness (50), as well as in rates of suicide disclosure (51). Future research should assess whether demographic factors moderate the findings. Participants were outpatients and the majority were not at acute risk for suicide, and these findings may not apply to more acutely ill people. Diagnostic groups were unbalanced, and small cell sizes in the diagnostic groups precluded subgroup analyses. Future research should explore how diagnosis may influence the likelihood of disclosing future suicidal thoughts. Our study did not assess other factors that may contribute to a lower likelihood of disclosing future suicidal thoughts to close others, such as internalized stigma, shame, or distrust, which may be important considerations for future research (52). Although indirect effects can still be observed in the absence of a significant association between the independent variable and the dependent variable (53), exploratory path analyses within the present study were limited to models in which there was a significant association between the social network feature and likelihood to disclose suicidal thoughts to reduce the number of analyses. This approach can introduce data-driven selection bias, and results should be interpreted with caution.

Notwithstanding these limitations, our study used a social network approach to find that perceived burdensomeness and thwarted belongingness are associated with quality and functional social network indicators, and that belongingness in particular may play a unique and potentially explanatory role in the likelihood of disclosing future suicidal thoughts to others. Considering disclosure is a critical step in the process of initiating many if not most suicide prevention interventions (54), our findings suggest that thwarted belongingness is a target for increasing disclosure likelihood, extending its original application and conceptualization as relating to suicidal desire. Influences on disclosure may be one pathway of how thwarted belongingness increases risk for suicide, and social network measures may help to specify how strong social connections can promote disclosure of suicidal thoughts.

## Data Availability

The raw data supporting the conclusions of this article will be made available by the authors, without undue reservation.
