# Supplementary material for: The role of perceived burdensomeness and thwarted belongingness in suicide disclosure likelihood: a social network perspective
Source: Front Psychiatry. 2026 Aug 11;17:1877025. doi: 10.3389/fpsyt.2026.1877025 (PMC13503556; doi:10.3389/fpsyt.2026.1877025)
Supplement: Supplementary file 1 [file Supplementaryfile1.docx]

**Supplement 1.** Path model coefficients for perceived burdensomeness (PB) models after adjusting for depression

| ***Predictor*** | ***Path*** | ***Standard Estimate*** | ***SE*** | ***p*** |
| --- | --- | --- | --- | --- |
| **Satisfaction** | a: satisfaction -> PB | -0.149 | 1.274 | .287 |
|  | b: PB -> disclosure likelihood | -0.303 | 0.023 | .013 |
|  | c’: satisfaction -> disclosure likelihood (adjusting for PB) | 0.074 | 0.242 | .598 |
|  | Indirect effect, $a\times b$ | 0.045 | 0.070 | .270 |
| **Practical support** | a: practical support -> PB | -0.339 | 0.932 | .006 |
|  | b: PB -> disclosure likelihood | -0.245 | 0.027 | .083 |
|  | c’: practical support -> disclosure likelihood (adjusting for PB) | 0.171 | 0.239 | .305 |
|  | Indirect effect, $a\times b$ | 0.083 | 0.076 | .118 |

**Supplement 2.** Path model coefficients for perceived burdensomeness (PB) models after adjusting for presence of current suicidal ideation

| ***Predictor*** | ***Path*** | ***Standard Estimate*** | ***SE*** | ***p*** |
| --- | --- | --- | --- | --- |
| **Satisfaction** | a: satisfaction -> PB | -0.300 | 1.402 | .052 |
|  | b: PB -> disclosure likelihood | -0.271 | 0.020 | .011 |
|  | c’: satisfaction -> disclosure likelihood (adjusting for PB) | 0.095 | 0.228 | .474 |
|  | Indirect effect, $a\times b$ | 0.081 | 0.083 | .095 |
| **Practical support** | a: practical support -> PB | -0.415 | 1.047 | .003 |
|  | b: PB -> disclosure likelihood | -0.222 | 0.021 | .045 |
|  | c’: practical support -> disclosure likelihood (adjusting for PB) | 0.184 | 0.214 | .220 |
|  | Indirect effect, $a\times b$ | 0.092 | 0.080 | .103 |

**Supplement 3.** Path model coefficients for thwarted belongingness (TB) models after adjusting for depression

| ***Predictor*** | ***Path*** | ***Standard Estimate*** | ***SE*** | ***p*** |
| --- | --- | --- | --- | --- |
| **Closeness** | a: closeness -> TB | -0.458 | 2.180 | <.001 |
|  | b: TB -> disclosure likelihood | -0.418 | 0.015 | .002 |
|  | c’: closeness -> disclosure likelihood (adjusting for TB) | 0.195 | 0.322 | .192 |
|  | Indirect effect, $a\times b$ | 0.191 | 0.181 | .023 |
| **Satisfaction** | a: satisfaction -> TB | -0.617 | 2.051 | <.001 |
|  | b: TB -> disclosure likelihood | -0.684 | 0.016 | <.001 |
|  | c’: satisfaction -> disclosure likelihood (adjusting for TB) | -0.286 | 0.319 | .121 |
|  | Indirect effect, $a\times b$ | 0.423 | 0.257 | .004 |
| **Practical support** | a: practical support -> TB | -0.426 | 1.551 | <.001 |
|  | b: TB -> disclosure likelihood | -0.484 | 0.014 | <.001 |
|  | c’: practical support -> disclosure likelihood (adjusting for TB) | 0.054 | 0.218 | .720 |
|  | Indirect effect, $a\times b$ | 0.206 | 0.117 | .011 |
| **Emotional support** | a: emotional support -> TB | -0.593 | 1.619 | <.001 |
|  | b: TB -> disclosure likelihood | -0.276 | 0.018 | .093 |
|  | c’: emotional support -> disclosure likelihood (adjusting for TB) | 0.351 | 0.261 | .040 |
|  | Indirect effect, $a\times b$ | 0.164 | 0.151 | .096 |

**Supplement 4.** Path model coefficients for thwarted belongingness (TB) models after adjusting for presence of current suicidal ideation

| ***Predictor*** | ***Path*** | ***Standard Estimate*** | ***SE*** | ***p*** |
| --- | --- | --- | --- | --- |
| **Closeness** | a: closeness -> TB | -0.445 | 2.315 | <.001 |
|  | b: TB -> disclosure likelihood | -0.433 | 0.014 | <.001 |
|  | c’: closeness -> disclosure likelihood (adjusting for TB) | 0.120 | 0.315 | .411 |
|  | Indirect effect, $a\times b$ | 0.193 | 0.181 | .022 |
| **Satisfaction** | a: satisfaction -> TB | -0.688 | 1.812 | <.001 |
|  | b: TB -> disclosure likelihood | -0.650 | 0.015 | <.001 |
|  | c’: satisfaction -> disclosure likelihood (adjusting for TB) | -0.258 | 0.281 | .113 |
|  | Indirect effect, $a\times b$ | 0.447 | 0.230 | .001 |
| **Practical support** | a: practical support -> TB | -0.488 | 1.561 | <.001 |
|  | b: TB -> disclosure likelihood | -0.449 | 0.013 | <.001 |
|  | c’: practical support -> disclosure likelihood (adjusting for TB) | 0.059 | 0.195 | .665 |
|  | Indirect effect, $a\times b$ | 0.219 | 0.119 | .008 |
| **Emotional support** | a: emotional support -> TB | -0.641 | 1.737 | <.001 |
|  | b: TB -> disclosure likelihood | -0.325 | 0.016 | .026 |
|  | c’: emotional support -> disclosure likelihood (adjusting for TB) | 0.269 | 0.255 | .105 |
|  | Indirect effect, $a\times b$ | 0.208 | 0.147 | .029 |

**ChatGPT**

Initial prompt: I am looking to do a mediation analysis. The data has multiple levels at the independent variable and the dependent variable but not the mediator (between persons data). how would I do this in R?

Response:

\# Packages

library(lme4)

library(dplyr)

\# Assume your long data: id (person), time (occasion), X, M (between-person), Y

\# 1) Decompose X (and optionally Y) into within- and between-person parts

d2 <- your_data %>%

group_by(id) %>%

mutate(

X_pm = mean(X, na.rm = TRUE), # person-mean (between-person X)

X_wp = X - X_pm	# within-person centered X

) %>%

ungroup()

\# 2) Path a model (between-person): M ~ X_pm

\#	Because M is L2, you can just use lm() with one row per person OR use all rows (same result for fixed effects).

\#	Safer: collapse to one row per person for clarity.

d_person <- d2 %>%

group_by(id) %>%

summarise(X_pm = first(X_pm), M = first(M), .groups = "drop")

m_a <- lm(M ~ X_pm, data = d_person)

a <- coef(m_a)[["X_pm"]]

\# 3) Path b/c' model (mixed): Y ~ X_wp + X_pm + M + random effects

\#	Include a random intercept for id, and (optionally) a random slope for X_wp if it varies across people.

m_b <- lmer(Y ~ X_wp + X_pm + M + (1 + X_wp | id), data = d2, REML = FALSE)

b	<- fixef(m_b)[["M"]]

c_wp <- fixef(m_b)[["X_wp"]] # within-person direct effect (not part of indirect)

c_bp <- fixef(m_b)[["X_pm"]] # between-person direct effect (c′_between)

indirect <- a * b # between-person indirect effect via M

direct_between <- c_bp

total_between <- direct_between + indirect

summary(m_a)

summary(m_b)

indirect

total_between
